# Identifying homologous recombination deficiency in breast cancer: genomic instability score distributions differ among breast cancer subtypes

**DOI:** 10.1007/s10549-023-07046-3

**Published:** 2023-08-17

**Authors:** Lauren Lenz, Chris Neff, Cara Solimeno, Elizabeth S. Cogan, Vandana G. Abramson, Judy C. Boughey, Carla Falkson, Matthew P. Goetz, James M. Ford, William J. Gradishar, Rachel C. Jankowitz, Virginia G. Kaklamani, P. Kelly Marcom, Andrea L. Richardson, Anna Maria Storniolo, Nadine M. Tung, Shaveta Vinayak, Darren R. Hodgson, Zhongwu Lai, Simon Dearden, Bryan T. Hennessy, Erica L. Mayer, Gordon B. Mills, Thomas P. Slavin, Alexander Gutin, Roisin M. Connolly, Melinda L. Telli, Vered Stearns, Jerry S. Lanchbury, Kirsten M. Timms

**Affiliations:** 1 Myriad Genetics, Inc, 320 Wakara Way, Salt Lake City, UT 84108 USA; 2 Vanderbilt University Medical Center, Nashville, TN USA; 3 Mayo Clinic, Rochester, MN USA; 4 University of Rochester Medical Center, Rochester, NY USA; 5 Stanford University School of Medicine, Stanford, CA USA; 6 Northwestern University, Chicago, IL USA; 7 University of Pennsylvania, Philadelphia, PA USA; 8 University of Texas Health Science Center at San Antonio, San Antonio, TX USA; 9 Duke University, Durham, NC USA; 10 Sidney Kimmel Comprehensive Cancer Center, Johns Hopkins School of Medicine, Baltimore, MD USA; 11 Melvin and Bren Simon Comprehensive Cancer Center, Indiana University School of Medicine, Indianapolis, IN USA; 12 Beth Israel Deaconess Medical Center, Boston, MA USA; 13 University of Washington, Seattle, WA USA; 14 Fred Hutchinson Cancer Research Center, 15. AstraZeneca, Seattle, WA USA; 15 AstraZeneca, Cambridge, UK; 16 AstraZeneca, Boston, MA USA; 17 Royal College of Surgeons in Ireland, Dublin, Ireland; 18 Dana-Farber Cancer Institute, Boston, MA USA; 19 Harvard Medical School, Boston, MA USA; 20 Oregon Health & Science University, Portland, OR USA; 21 Cancer Research @UCC, University College Cork, Cork, Ireland

**Keywords:** DNA damage, Genomic instability, Homologous recombination deficiency, Breast cancer, Tumor biomarker

## Abstract

**Purpose:**

A 3-biomarker homologous recombination deficiency (HRD) score is a key component of a currently FDA-approved companion diagnostic assay to identify HRD in patients with ovarian cancer using a threshold score of ≥ 42, though recent studies have explored the utility of a lower threshold (GIS ≥ 33). The present study evaluated whether the ovarian cancer thresholds may also be appropriate for major breast cancer subtypes by comparing the genomic instability score (GIS) distributions of *BRCA1/2*-deficient estrogen receptor–positive breast cancer (ER + BC) and triple-negative breast cancer (TNBC) to the GIS distribution of *BRCA1/2-*deficient ovarian cancer.

**Methods:**

Ovarian cancer and breast cancer (ER + BC and TNBC) tumors from ten study cohorts were sequenced to identify pathogenic *BRCA1/2* mutations, and GIS was calculated using a previously described algorithm. Pathologic complete response (pCR) to platinum therapy was evaluated in a subset of TNBC samples. For TNBC, a threshold was set and threshold validity was assessed relative to clinical outcomes.

**Results:**

A total of 560 ovarian cancer, 805 ER + BC, and 443 TNBC tumors were included. Compared to ovarian cancer, the GIS distribution of *BRCA1/2*-deficient samples was shifted lower for ER + BC (p = 0.015), but not TNBC (p = 0.35). In the subset of TNBC samples, univariable logistic regression models revealed that GIS status using thresholds of ≥ 42 and ≥ 33 were significant predictors of response to platinum therapy.

**Conclusions:**

This study demonstrated that the GIS thresholds used for ovarian cancer may also be appropriate for TNBC, but not ER + BC. GIS thresholds in TNBC were validated using clinical response data to platinum therapy.

**Supplementary Information:**

The online version contains supplementary material available at 10.1007/s10549-023-07046-3.

## Introduction

Precision medicine can have important implications for management and treatment of individuals with cancer. Aggressive chemotherapy regimens can have intolerable side effects and carry the risk of weakening organ or immune functions without clinical benefit; even targeted anti-cancer drugs can damage healthy tissue. Identifying the presence of biomarkers that indicate whether tumors are likely to be sensitive to specific chemotherapies can optimize treatment selection, thus increasing the likelihood that patients will receive tolerable and effective treatment regimens. DNA-damaging agents are a targeted treatment which can exploit existing DNA deficiencies in tumors by inhibiting or overwhelming repair pathways [1]. Consistent with this concept, patients who have tumors with homologous recombination (HR) deficiency (HRD) may benefit from treatment with platinum and poly (ADP-ribose) polymerase (PARP) inhibitors [2–5].

Several markers of defects in DNA repair can be used to identify HRD, including the presence of germline or somatic pathogenic variants in *BRCA1/2* and other genes involved in HR [6]. Individual measures of genomic instability, such as loss of heterozygosity (LOH), have also been utilized to determine tumor HRD status [7]. A 3-biomarker HRD signature assay was previously developed as a more robust way to measure HRD [8]. The test produces a combined genomic instability score (GIS) based on LOH, telomeric-allelic imbalance (TAI), and large-scale state transitions (LST) [8]. This test provides a comprehensive measure of tumor HRD, beyond what is captured by genetic deficiencies and/or a single measure of genomic instability (i.e., LOH) [7, 8]. Higher GIS is associated with treatment response to platinum-based therapies and PARP inhibitors, and GIS assessment is part of a United States Food and Drug Administration (FDA)–approved companion diagnostic for patients with ovarian cancer who may be eligible for PARP inhibitor treatment [9–14].

Currently, the 3-biomarker signature assay is FDA-approved to identify HRD in patients with ovarian cancer using a GIS threshold of ≥ 42. This threshold was determined using ovarian and breast cancer tumor samples, and was set as the 5th percentile of scores in *BRCA*-deficient tumors [11]. Tumors with mutations in *BRCA1/2* are likely to have HRD. Therefore, the GIS distribution in known *BRCA1/2-*deficient samples can be used to set thresholds. Recently, a lower threshold of ≥ 33 set at the 1st percentile of scores in *BRCA*-deficient tumors in ovarian and breast cancer has been explored; this threshold was significantly associated with improved outcomes after platinum-based treatment in ovarian cancer [9, 10, 15]. However, the GIS distribution may vary between different cancers and even between different cancer subtypes due to differences in disease pathology. Therefore, determining an optimal GIS threshold for different types of HRD tumors is important. While current evidence suggests that a threshold of ≥ 33 may be the most appropriate cutoff for ovarian cancer, it is unclear whether this recommendation should be extended to breast cancer and to distinct breast cancer subtypes.

The present study evaluated whether a GIS threshold of ≥ 33 is also appropriate for two major breast cancer subtypes: estrogen receptor–positive (ER+) breast cancer (ER + BC) and triple-negative breast cancer (TNBC). To evaluate this, GIS distributions of *BRCA1/2*-deficient ER + BC and TNBC were assessed and compared to the GIS distribution of *BRCA1/2*-deficient ovarian cancer. Clinical outcomes were available for a subset of TNBC samples, allowing a potential GIS threshold to be set and to evaluate the ability of this potential GIS threshold to predict response to platinum therapy.

## Methods

### Tumor samples

This retrospective study assessed ovarian and breast cancer tumors from ten individual study cohorts: Hennessy et al. [16], The Cancer Genome Atlas (TCGA) Research Network, [17, 18] NCT01372579, [19] NCT00148694/NCT00580333, [11] PrECOG 0105, [11] Timms et al., [8] TBCRC008, [20] TBCRC030, [21] and the OlympiAD trial [22]. All tumors with a known GIS from patients with ovarian cancer, ER + BC, or TNBC were selected for inclusion in the current analysis. Tumors with a known GIS from patients with ER-negative breast cancer were excluded from the analysis. Additional details on patient and specimen characteristics, inclusion and exclusion criteria, any treatments received, patient follow-up, and the time period of case collection are described, as applicable, in previous publications for the individual study cohorts. All included samples were obtained under protocols approved by an Institutional Review Board [8, 11, 16–22]. REMARK reporting guidelines have been followed as applicable [23].

### MyChoice testing

MyChoice testing (Myriad Genetics) was performed to determine somatic *BRCA1/2* status and GIS. Public TCGA data were downloaded from the Cancer Genomics Hub and run through MyChoice software, as previously described [24]. For all other specimens, MyChoice CDx testing was performed at the central Myriad Genetics reference laboratory at the time of the initial investigation [8, 11, 16, 19–22] following previously published methods [11, 25]. Details of the test, including test kit contents, requirements for biological specimens, test results and interpretation, and performance characteristics are provided in the technical specifications document [26].

### BRCA1/2 sequencing

Gene mutation detection for *BRCA1/2* and single-nucleotide polymorphism whole-genome analysis were performed using a custom hybridization capture method, as described previously [25]. Pathogenic *BRCA* mutation status was defined as a deleterious or suspected deleterious mutation in *BRCA1* or *BRCA2*, regardless of heterozygosity. *BRCA* wildtype (*BRCA*wt) refers to a sample with no deleterious or suspected deleterious mutation in *BRCA1* or *BRCA2. BRCA* deficiency was defined as loss of function resulting from a germline or somatic deleterious or suspected deleterious variant in *BRCA1* or *BRCA2* with LOH in the affected gene, or by multiple deleterious or suspected deleterious mutations in the same *BRCA* gene. *BRCA*-intact refers to a sample that is not *BRCA1/2* deficient, regardless of *BRCA* mutation status.

### Genomic instability score

GIS was calculated using an algorithm that combines measures of LOH, TAI, and LST, as previously described [25]. Binary GIS status was determined based on whether GIS scores were above or below a threshold of ≥ 33 or ≥ 42.

### Pathologic complete response

Pathologic complete response (pCR) to preoperative chemotherapy was available for TNBC samples from five cohorts (NCT01372579, [19] NCT00148694/NCT00580333, [11] PrECOG 0105, [11] TBCRC008, [20] and TBCRC030 [21]). pCR status was not available for ER + samples. In some studies, residual cancer burden (RCB) [27] was used and pCR status was not available. Patients with data on RCB [11, 19–21] after treatment with platinum therapy were dichotomized into those with complete response (RCB-0) and those with incomplete response (RCB-I/II/III). Patients with RCB-0 who did not receive crossover treatment prior to surgery and who did not exit treatment due to progression or toxicity were considered to have achieved pCR.

### Statistics

Two-sided Kolmogorov-Smirnov tests were used to compare GIS distributions in *BRCA1/2*-deficient ER + BC samples by human epidermal growth factor receptor 2 (HER2) status. Additionally, the GIS distributions of *BRCA1/2*-deficient ER + BC and TNBC samples were compared to that of *BRCA1/2*-deficient ovarian cancer samples.

Binomial logistic regression was used to measure the ability of binary GIS status (i.e., scores above or below the threshold) to predict pCR status in TNBC tumors. Odds ratios (ORs) with 95% profile likelihood confidence intervals (CIs) and partial likelihood ratio test p-values were reported. Sensitivity, specificity, positive predictive value (PPV), and negative predictive value (NPV), were calculated by comparing binary GIS status and binary pCR status, where a pCR event with a GIS above the threshold was considered a true positive. Univariable three-parameter logistic regression models optimized for the upper bound, slope, and midpoint were used to estimate the probability of pCR for each GIS value.

All p-values were considered significant at the α = 0.05 level.

## Results

### Ovarian cancer tumors

**Fig. 1:**
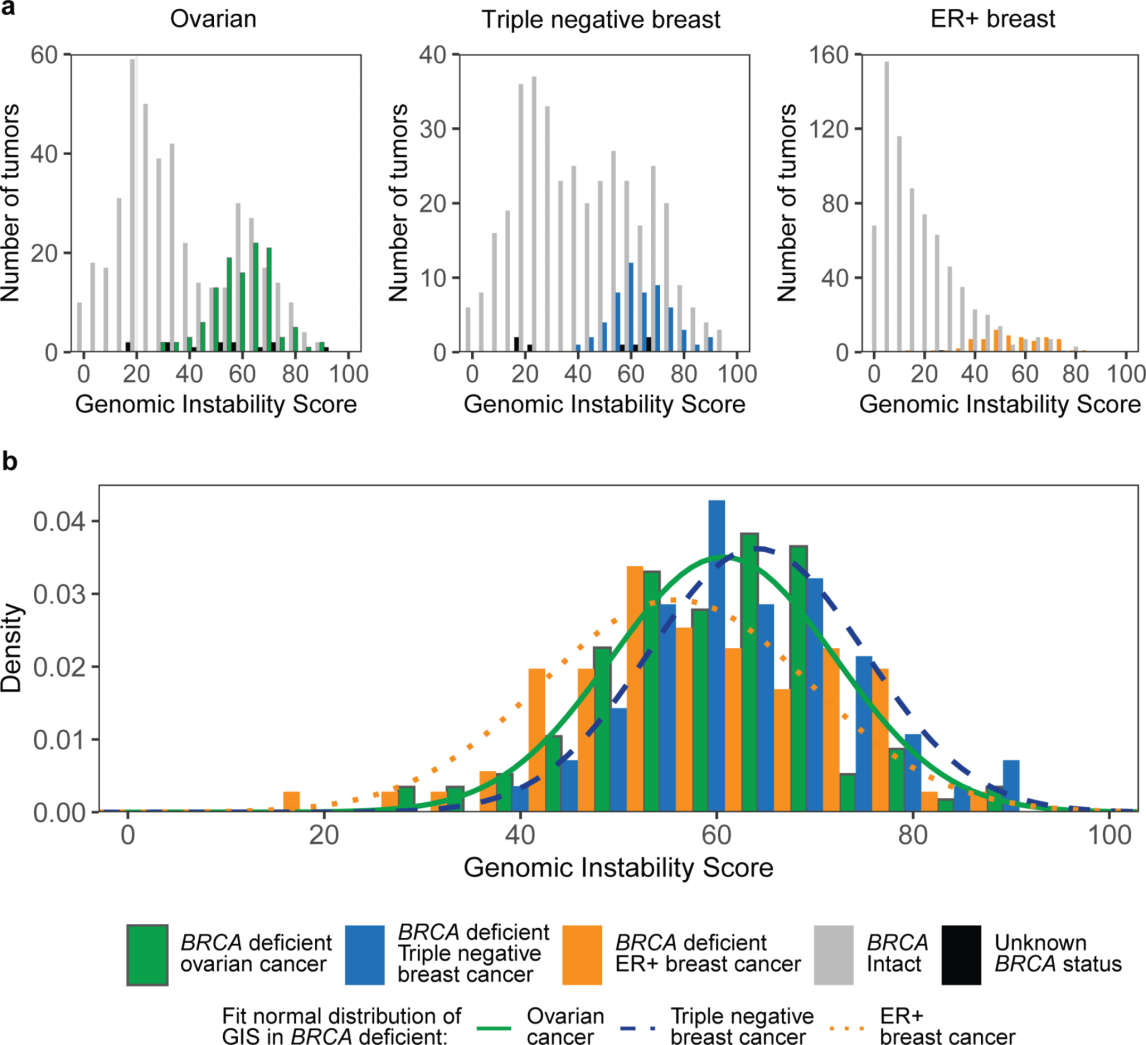
Distribution of GIS by cancer type and *BRCA* status. (**a**) The distribution of GIS for *BRCA1/2*-deficient and *BRCA*-intact tumors in ovarian cancer, TNBC, and ER + BC. (**b**) The distribution of GIS for *BRCA1/2-*deficient tumors fit to a normal distribution for ovarian cancer, TNBC, and ER + BC

A total of 560 ovarian cancer tumors from two cohorts (Hennessy et al. [16], and The Cancer Genome Atlas Network– Ovarian [18]) were included, 20.5% of which were known to be *BRCA1/2-*deficient (N = 115/560; Table 1). Among *BRCA1/2-*deficient samples, 67.8% (N = 78/115) had a pathogenic mutation in *BRCA1*, 31.3% (N = 36/115) had a pathogenic mutation in *BRCA2*, and 0.9% (N = 1/115) had a pathogenic mutation in both *BRCA1* and *BRCA2*. The GIS distributions are shown in Fig. 1a for *BRCA1/2-*deficient and *BRCA-*intact tumors and **Supplemental Fig. 1** for *BRCA1-*deficient and *BRCA2*-deficient tumors. The median GIS was 62 in *BRCA1/*2-deficient ovarian cancer tumors and 31 in *BRCA*-intact tumors (Table 1, **Supplemental Fig. 2**). In this analysis, the GIS distribution of *BRCA1/2-*deficient ovarian cancer samples was used as a comparator to evaluate GIS distributions in *BRCA1/2-*deficient ER + BC and TNBC samples.

**Table 1:** Summary of analysis cohorts

	Ovarian Cancer Tumors N = 560 N (%) or Median (IQR)	ER + BC Tumors N = 805 N (%) or Median (IQR)	TNBC Tumors N = 443 N (%) or Median (IQR)	TNBC Clinical Validation Tumors N = 211 N (%) or Median (IQR)
**Cohort, n (%)**				
Timms et al.	0 (0%)	112 (13.9%)	55 (12.4%)	0 (0%)
Hennessy et al.	135 (24.1%)	0 (0%)	0 (0%)	0 (0%)
TBCRC030	0 (0%)	0 (0%)	107 (24.2%)	56 (26.5%)
TBCRC008	0 (0%)	25 (3.1%)	18 (4.1%)	17 (8.1%)
NCT01372579	0 (0%)	0 (0%)	26 (5.9%)	26 (12.3%)
OlympiAD	0 (0%)	52 (6.5%)	0 (0%)	0 (0%)
The Cancer Genome Atlas Network – Breast	0 (0%)	614 (76.3%)	119 (26.9%)	0 (0%)
The Cancer Genome Atlas Network – Ovarian	425 (75.9%)	0 (0%)	0 (0%)	0 (0%)
NCT00148694/ NCT00580333	0 (0%)	0 (0%)	51 (11.5%)	48 (22.7%)
PrECOG 0105	0 (0%)	2 (0.2%)	67 (15.1%)	64 (30.3%)
**Patient Age, median (IQR)**	59 (51, 69)	58 (48, 67)	52 (43, 60)	50 (42, 59)
**Patient Sex, n (%)**				
Female	560 (100%)	689 (85.6%)	410 (92.6%)	211 (100%)
Male	0 (0%)	11 (1.4%)	0 (0%)	0 (0%)
Unknown	0 (0%)	105 (13.0%)	33 (7.4%)	0 (0%)
***BRCA*** **Mutation Status, n (%)**				
*BRCA1*	79 (14.1%)	31 (3.9%)	49 (11.1%)	27 (12.8%)
*BRCA2*	38 (6.8%)	52 (6.5%)	10 (2.3%)	7 (3.3%)
*BRCA1* and *BRCA2*	1 (0.2%)	0 (0%)	2 (0.5%)	1 (0.5%)
*BRCA*wt	332 (59.3%)	721 (89.6%)	376 (84.9%)	171 (81.0%)
Unknown	110 (19.6%)	1 (0.1%)	6 (1.4%)	5 (2.4%)
***BRCA*** **Deficiency Status, n (%)**				
*BRCA1*	78 (13.9%)	29 (3.6%)	47 (10.6%)	26 (12.3%)
*BRCA2*	36 (6.4%)	42 (5.2%)	8 (1.8%)	6 (2.8%)
*BRCA1* and *BRCA2*	1 (0.2%)	0 (0%)	1 (0.2%)	0 (0%)
*BRCA* intact	432 (77.1%)	733 (91.1%)	380 (85.8%)	173 (82.0%)
Unknown	13 (2.3%)	1 (0.1%)	7 (1.6%)	6 (2.8%)
**GIS, median (IQR)**				
All tumors	39 (23, 62)	16 (7, 31)	46 (26, 64)	51 (28, 66)
*BRCA1/2* deficient	62 (54, 68)	56 (47, 67)	64 (57, 70)	63 (57, 69)
*BRCA* intact	31 (20, 56)	14 (6, 26)	40 (24, 60)	46 (26, 66)
**pCR Status, n (%)**				
pCR	-	-	-	55 (26.1%)
No pCR	-	-	-	156 (73.9%)

Abbreviations:

*BRCA*wt, *BRCA* wildtype; ER+, estrogen receptor positive; GIS, genomic instability score; IQR, interquartile range; pCR, pathologic complete response; TNBC, triple negative breast cancer.

Age was not readily accessible for a total of 16 patients: 11/560 (2.0%) ovarian samples; 3/805 (0.4%) ER + samples; and 2/443 (0.5%) TNBC samples, including none of the TNBC clinical validation tumors.

### ER + BC tumors

A total of 805 ER + BC tumors were included from five cohorts (The Cancer Genome Atlas Network – Breast, [17] PrECOG 0105, [11] Timms et al., [8] TBCRC008, [20] and the OlympiAD trial [22]; Table 1). Of these, 579 were ER + HER2-, 174 were ER + HER2+, and 52 were ER + with unknown HER2 status. To determine whether it would be appropriate to combine all ER + BC tumors, the GIS distributions of *BRCA1/2-*deficient tumors for ER + HER2- (N = 60) and ER + HER2+ (N = 10) were compared. No significant differences were observed between GIS distributions of ER + HER2- and ER + HER2 + *BRCA1/2-*deficient tumors (p = 0.80; **Supplemental Fig. 3**). However, with only ten *BRCA1/2-*deficient ER + HER2 + samples, this comparison is underpowered. In the future, when more ER + HER2 + samples are available, ER + HER2- and ER + HER2 + samples may be compared more rigorously. In these analyses, all ER + samples were analyzed together to increase statistical power.

Among all ER + BC tumors, 8.8% (N = 71/805; N = 60 ER + HER2-, N = 10 ER + HER+, and N = 1 ER + HER2 status unknown) were *BRCA1/2-*deficient; of those, 40.8% (N = 29/71) had a pathogenic mutation in *BRCA1*, and 59.2% (N = 42/71) had a pathogenic mutation in *BRCA2* (see **Supplemental Fig. 1** for GIS distributions of *BRCA1*-deficient and *BRCA2-*deficient samples). The GIS distributions of *BRCA1/2-*deficient and *BRCA-*intact tumors are shown in Fig. 1a. The median GIS was 56 in *BRCA1/2-*deficient ER + BC tumors and 14 in *BRCA*-intact tumors (Table 1, **Supplemental Fig. 2**). A significant difference was observed between the GIS distributions for *BRCA1/2*-deficient ER + BC tumors and ovarian cancer tumors (p = 0.015; Fig. 1b), indicating that a separate threshold should be established for ER + BC tumors. A potential GIS threshold will be established in a future study when clinical outcomes for ER + BC tumors treated with platinum or other DNA-damaging agents are available.

### TNBC tumors

A total of 443 TNBC tumors were included from seven cohorts (The Cancer Genome Atlas Network – Breast, [17] NCT01372579, [19] NCT00148694/NCT00580333, [11] PrECOG 0105, [11] Timms et al., [8] TBCRC008, [20] and TBCRC030 [21]; Table 1). Among the 56 (12.6%) *BRCA1/2-*deficient TNBC tumors, 47 (83.9%) had a pathogenic mutation in *BRCA1*, 8 (14.3%) had a pathogenic mutation in *BRCA2*, and 1 (1.8%) had pathogenic mutations in both *BRCA1* and *BRCA2* (see **Supplemental Fig. 1** for GIS distributions of *BRCA1*-deficient and *BRCA2-*deficient samples). The GIS distributions of *BRCA1/2-*deficient and *BRCA-*intact tumors are shown in Fig. 1a. The median GIS was 64 in *BRCA1/2-*deficient TNBC tumors and 40 in *BRCA*-intact tumors (Table 1, **Supplemental Fig. 2**). When comparing the GIS distributions of *BRCA1/2*-deficient samples, TNBC tumors were significantly different from ER + BC tumors (p < 0.001; Fig. 1b), but not significantly different from ovarian cancer tumors (p = 0.35; Fig. 1b). This indicates that the same thresholds used for ovarian cancer tumors may also be appropriate for TNBC tumors.

### Clinical validation of thresholds in TNBC

**Fig. 2:**
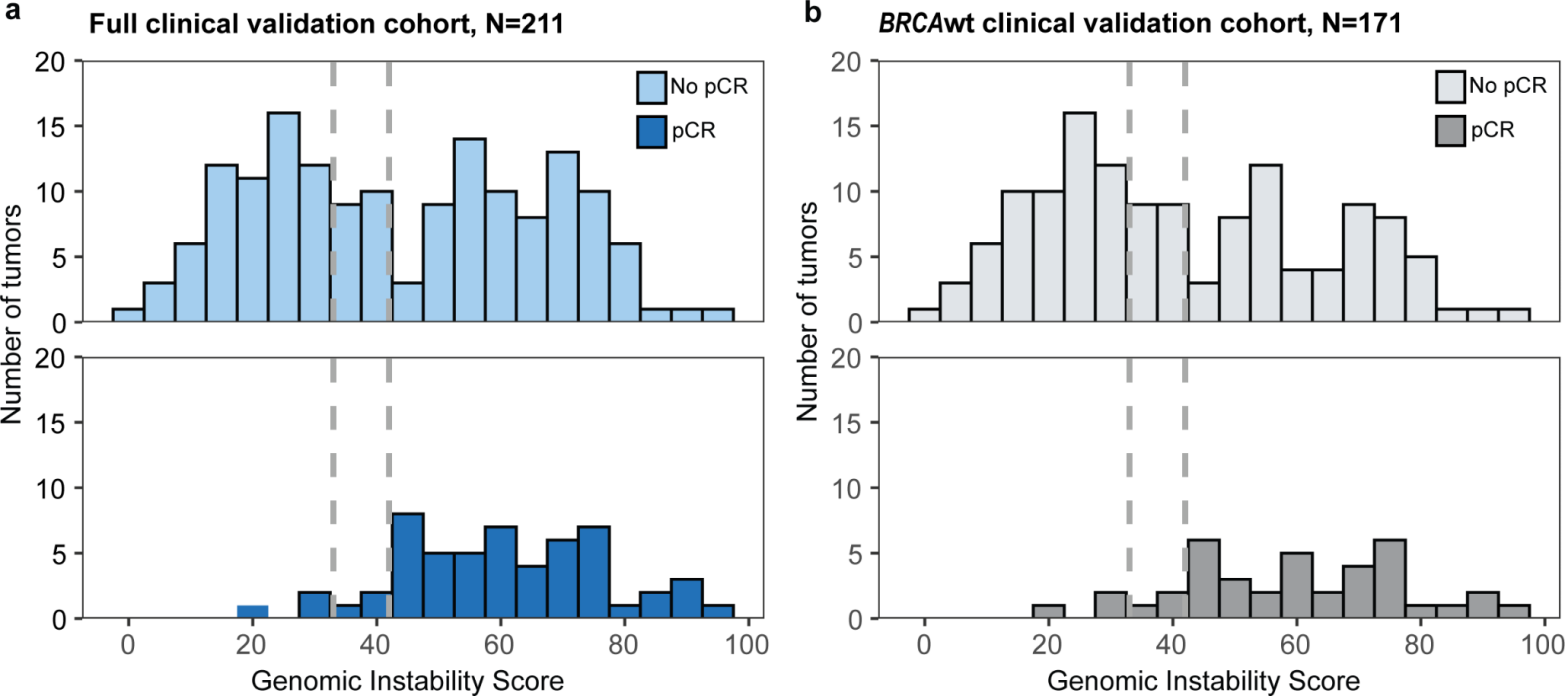
Distribution of GIS by pCR status for TNBC in (**a**) the full clinical validation cohort, and (**b**) the *BRCA*wt clinical validation cohort. Samples are stratified based on whether pCR was achieved (“pCR” vs. “No pCR”)

GIS thresholds of ≥ 42 and ≥ 33 have been previously validated in patients with ovarian cancer [9–11, 15]. Because the GIS distributions in *BRCA1/2*-deficient ovarian and TNBC samples were similar, the thresholds used for ovarian cancer were applied to the TNBC samples in this study. The TNBC clinical validation cohort (samples from the following preoperative trials: NCT01372579, [19] NCT00148694/NCT00580333, [11] PrECOG 0105, [11] TBCRC008, [20] and TBCRC030 [21]) included 211 platinum-treated samples (N = 55 with pCR), 171 of which were *BRCA*wt tumors (N = 39 with pCR)) (Table 1**)**. GIS distributions for all TNBC clinical validation samples (full clinical validation cohort) and for the subset of *BRCA*wt samples (*BRCA*wt clinical validation cohort) are summarized by binary pCR status (i.e., pCR vs. no pCR) in Fig. 2.

Univariable logistic regression models were used to evaluate the ability of GIS status, using thresholds of ≥ 33 and ≥ 42, to predict binary pCR status in both the full clinical validation cohort and in the *BRCA*wt clinical validation cohort. In both cohorts, GIS status using thresholds of ≥ 33 and ≥ 42 were significant predictors of pCR. Compared to the GIS threshold status of ≥ 42, the GIS threshold status of ≥ 33 resulted in a larger effect size in both the full clinical validation cohort (GIS ≥ 33: OR 11.1, 95% CI 3.9–47.1, p = 2.2 × 10^− 7^; GIS ≥ 42: OR 8.2, 95% CI 3.5–22.3, p = 5.6 × 10^− 8^) and the *BRCA*wt clinical validation cohort (GIS ≥ 33: OR 9.4, 95% CI 3.2–40.4, p = 5.6 × 10^− 6^; GIS ≥ 42: OR 7.0, 95% CI 2.9–19.6, p = 3.0 × 10^− 6^). A comparison of ORs from univariable logistic regression models evaluating the ability of GIS status set at a range of thresholds to predict pCR is shown in **Supplemental Fig. 4**.

To evaluate the ability of the GIS status using thresholds of ≥ 33 and ≥ 42 to predict pCR, a bivariable logistic regression model was run with both GIS threshold statuses as binary variables. In the full clinical validation cohort, the GIS threshold status of ≥ 42 was significant (OR 3.6, 95% CI 1.1–15.8, p = 0.03), while the GIS threshold status of ≥ 33 was not (OR 3.6, 95% CI 0.6–21.0, p = 0.15). In the same model fit in the *BRCA*wt clinical validation cohort, neither of the GIS threshold statuses were significant (GIS ≥ 33: OR 3.6, 95% CI 0.6–21.3, p = 0.15; GIS ≥ 42: OR 3.0, 95% CI 0.9–13.7, p = 0.07). These results demonstrate that the GIS threshold status of ≥ 42 adds significant information to the GIS threshold status of ≥ 33 in the full clinical validation cohort.

Sensitivity, specificity, PPV, and NPV for the pre-specified thresholds are reported in Table 2 for GIS thresholds of ≥ 33 and ≥ 42.

**Table 2:** Sensitivity, specificity, PPV, and NPV of GIS thresholds to predict pCR in TNBC.

Threshold	Sensitivity	Specificity	PPV	NPV
Full clinical validation cohort				
GIS ≥ 33	0.945	0.391	0.354	0.953
GIS ≥ 42	0.891	0.500	0.386	0.929
*BRCA*wt clinical validation cohort				
GIS ≥ 33	0.923	0.439	0.327	0.951
GIS ≥ 42	0.846	0.561	0.363	0.925

Abbreviations: *BRCA*wt, *BRCA* wildtype; GIS, genomic instability score; NPV, negative predictive value; PPV, positive predictive value; TNBC, triple negative breast cancer

A high proportion of samples with pCR events had a GIS ≥ 33 in both the full clinical validation cohort (94.5%, N = 52/55) and the *BRCA*wt clinical validation cohort (92.3%, N = 36/39). The proportion of pCR events captured by the threshold decreased at the higher GIS threshold of ≥ 42 (full clinical validation cohort: 89.1%, N = 49/55; *BRCA*wt clinical validation cohort: 84.6%, N = 33/39); a GIS between 33 and 42 captured pCR events in an additional 5.5% of the full clinical validation cohort and 7.7% of the *BRCA*wt subset.

**Fig. 3:**
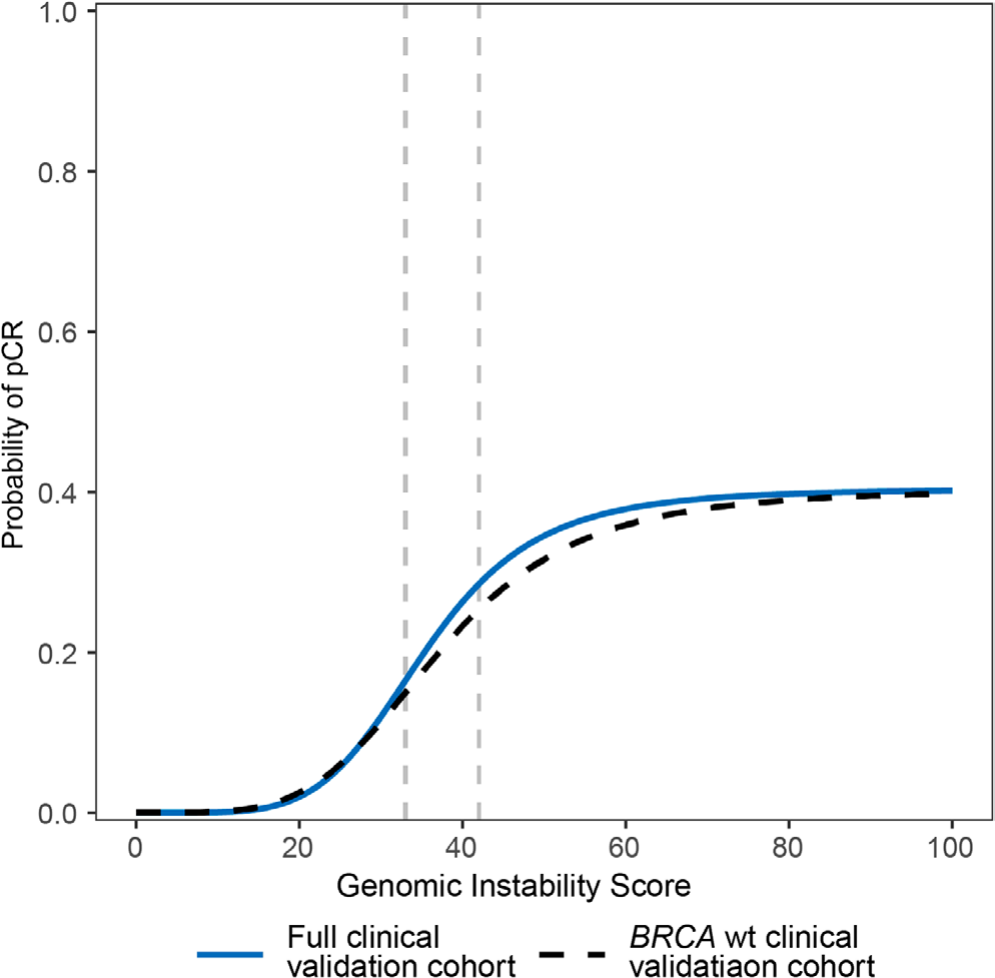
The probability of pCR in TNBC for a range of GIS from 3-parameter logistic regression models fit for the full clinical validation cohort (N = 211, solid line) and the *BRCA*wt clinical validation cohort (N = 171, dashed line). The vertical grey dashed lines represent potential thresholds of ≥ 33 and ≥ 42

The difference in utility between a threshold of ≥ 33 and ≥ 42 can also be characterized by the difference in probability of pCR as calculated by a three-parameter logistic regression with continuous GIS predicting binary pCR status (Fig. 3). In both the full clinical validation cohort and the *BRCA*wt clinical validation cohort, patients with GIS between 33 and 42 had an intermediate probability of pCR; a GIS threshold of ≥ 33 separated patients with a low probability of response from patients with a moderate to high probability of response. The opposite was true for the GIS threshold of ≥ 42, which would only identify patients with the highest likelihood of response.

## Discussion

In the present study, the GIS distributions of *BRCA1/2-*deficient tumors were evaluated for two different major breast cancer subtypes. The GIS distribution of *BRCA1/2-*deficient tumors for ER + BC was significantly different from the distribution for ovarian cancer, indicating that the GIS threshold used for ovarian cancer may not be appropriate for ER + BC. The GIS distribution for *BRCA1/2-*deficient TNBC tumors in this study was not statistically significantly different from ovarian cancer but was significantly different from ER + BC tumors. Additionally, the clinical validation analysis demonstrated the ability of the GIS ≥ 33 and ≥ 42 thresholds to predict platinum-based therapy pCR in a subset of the TNBC samples. Together, these findings highlight the importance of determining individual thresholds for different cancer lineages and for different cancer subtypes.

Compared to *BRCA1/2-*deficient ovarian cancer tumors, the GIS distribution was significantly different for *BRCA1/2-*deficient ER + BC tumors, but not TNBC tumors. This may not be surprising, given that there are known similarities in the molecular signatures of ovarian cancer and TNBC. For example, messenger RNA expression is similarly up- or down-regulated in some genes (e.g., *AKT3, CCNE1, MYC, RB1*) and high mutation rates in specific genes (e.g., *BRCA1*, *RB1*, *TP53*) are observed in both TNBC and ovarian cancer [28, 29]. Further, both TNBC and ovarian cancer are considered copy number-driven cancers [17, 18]. Patients with ovarian cancer and TNBC are also more likely to have mutations in *BRCA1* than *BRCA2*, [30, 31] while, the opposite is true for patients with ER + BC [32]. Differences in the underlying biology, and thus GIS, between pathogenic *BRCA1*-mutated and *BRCA2*-mutated tumors may at least partially explain the observed differences between GIS distributions for TNBC and ER + BC.

GIS thresholds of ≥ 33 and ≥ 42, set at the 1st and 5th percentile of *BRCA-*deficient tumors, respectively, have been validated previously in ovarian cancer [10, 12, 13, 15]. Therefore, both thresholds were evaluated in the TNBC clinical validation cohort. When evaluated in independent analyses, both the GIS threshold statuses of ≥ 33 and ≥ 42 were found to significantly predict pCR to platinum therapy, although a non-significantly larger effect size was observed for the GIS threshold status of ≥ 33 compared to ≥ 42 (OR 11.1 vs. 8.2). In a bivariable model that assessed the relationship between the two threshold statuses (i.e., evaluated whether one threshold added significant information to the other) in the full clinical validation cohort, the GIS threshold status of ≥ 42 was significant, while the GIS threshold status of ≥ 33 was not. In the *BRCA*wt clinical validation cohort, neither of the GIS threshold statuses in the bivariable model were found to be significant. While the analysis in the full clinical validation cohort indicated that the GIS threshold status of ≥ 42 added significant predictive information to the GIS threshold status of ≥ 33, the null findings in the *BRCA*wt analysis suggested that the two GIS threshold statuses had similar predictive value for pCR. The clinical significance of these inconsistent findings was unclear; therefore, sensitivity and specificity were evaluated to assess the clinical validity of the two thresholds.

In both the full clinical validation cohort and the *BRCA*wt clinical validation cohort, the GIS threshold of ≥ 42 had lower sensitivity, but higher specificity than the ≥ 33 threshold. When selecting a GIS threshold to identify patients who will benefit from DNA-damaging agents (e.g., platinum, PARP inhibitors), it is important to consider the appropriate balance of sensitivity and specificity. The GIS threshold of ≥ 42 will result in fewer false positives (i.e., fewer patients who will not benefit from treatment being categorized HRD-positive), but also will result in fewer true positives (i.e., fewer patients who will benefit from treatment being categorized as HRD-positive). Among the patients who achieved pCR to platinum therapy, 5.5% of patients in the full clinical validation cohort and 7.7% of patients in the *BRCA*wt cohort would not be identified as eligible for treatment using the threshold of ≥ 42. In clinical settings, it may be beneficial to utilize a lower threshold of ≥ 33 in order to maximize the identification of eligible patients given a paucity of alternative treatment choices. The decision to pursue treatment with DNA-damaging agents can then be considered on an individual basis, which may be dependent upon several clinical factors.

The balance of sensitivity and specificity should also be considered when selecting a GIS threshold for clinical trials. This is particularly relevant in cases where study eligibility criteria may influence the GIS distribution. For example, clinical trials that have enrollment criteria that enrich for patients with HR-deficient tumors (e.g., *BRCA1/2-*mutated tumors, high-grade and/or serous subtypes, platinum-sensitive tumors) will shift the distribution toward a higher GIS, as patients with pathogenic *BRCA1/2–*mutated tumors have higher GIS. A higher GIS threshold may appear appropriate based on high specificity alone, which may mean that fewer patients who will benefit from treatment will be categorized as HRD-positive. However, whether it may be appropriate to prioritize specificity or sensitivity could depend on the study population, or other clinical factors (e.g., first-line treatment, metastatic disease).

To date, most of the studies evaluating HRD status and clinical outcomes in breast cancer have used a threshold of ≥ 42 to identify HRD-positive tumors. Several single-arm studies have demonstrated that HRD-positive status is associated with improved clinical outcomes after platinum-based therapy in ovarian cancer [10, 15, 33] and TNBC [11, 19, 34]. However, in randomized trials, no association between HRD and chemotherapy benefit was observed [21, 35]. One study that evaluated platinum-based treatment in metastatic TNBC reported no association between HRD status and clinical outcomes [35]. In that study, HRD testing was performed on treatment-naïve tumor samples, and therefore it is possible that reversion of *BRCA* mutations could have precluded an association between HRD status and outcomes. However, it is also possible that an association between HRD status and outcomes would have been observed if the GIS threshold of ≥ 33 had been used.

One limitation of this study was the absence of clinical outcomes data for ER + BC; future studies will be needed to identify and validate potential thresholds. Additionally, the clinical outcomes evaluated for TNBC were limited to platinum-based therapy response. The thresholds discussed here should also be validated using other DNA-damaging agents (e.g., PARP inhibitors) in future studies. Further, the availability of data on receptor/molecular sub-types was limited in this study. It would be beneficial to compare additional tumor characteristics (e.g., Luminal A, Luminal B, HER2, etc.) in future studies to determine whether these thresholds should be broadly applied to all breast cancer sub-types.

The present study demonstrated that the optimal GIS threshold of ≥ 33 for ovarian cancer is also appropriate to predict platinum-therapy response for TNBC but may not be appropriate for ER + BC. Future studies evaluating the association between these thresholds and clinical outcomes will be required to demonstrate expanded clinical validity in response to other treatments, and in other breast cancer subtypes. The different GIS distributions observed in this study highlight the need for cancer-specific and cancer subtype–specific GIS thresholds. This will be especially important as evaluations of HRD to identify candidates for treatment with DNA-damaging agents become more commonly used in clinical practice and expand to different cancers.

## Electronic supplementary material

Below is the link to the electronic supplementary material.

Supplementary Material 1

## Data Availability

The datasets analyzed during the current study are not publicly available due to patient privacy but are available from the corresponding author on reasonable request.
