# Supplementary material for: Identifying homologous recombination deficiency in breast cancer: genomic instability score distributions differ among breast cancer subtypes
Source: Breast Cancer Res Treat. 2023 Aug 17;202(1):191–201. doi: 10.1007/s10549-023-07046-3 (PMC10504389; doi:10.1007/s10549-023-07046-3)
Supplement: Supplementary file 1 — Supplementary Material 1 [file 10549_2023_7046_MOESM1_ESM.docx]

**Supplemental Materials:** **Identifying homologous recombination deficiency in breast cancer: genomic instability score distributions differ among breast cancer subtypes**

Lauren Lenz, MS^1^; Chris Neff, BS^1^; Cara Solimeno, BS^1^; Elizabeth S. Cogan, PhD^1^; Vandana G. Abramson, MD^2^; Judy C. Boughey, MD^3^; Carla Falkson, MD^4^; Matthew P. Goetz, MD^3^; James M. Ford, MD^5^; William J. Gradishar, MD^6^; Rachel C. Jankowitz, MD^7^; Virginia G. Kaklamani, MD^8^; P. Kelly Marcom, MD^9^; Andrea L. Richardson, MD, PhD^10^; Anna Maria Storniolo, MD^11^; Nadine M. Tung, MD^12^; Shaveta Vinayak, MD, MS^13,14^; Darren R. Hodgson, PhD^15^; Zhongwu Lai, PhD^16^; Simon Dearden, MSc^15^; Bryan T. Hennessy, MD^17^; Erica L. Mayer, MD, MPH^18,19^; Gordon B. Mills, MD, PhD^20^; Thomas P. Slavin, MD^1^; Alexander Gutin, PhD^1^; Roisin M. Connolly, MD^21^; Melinda L. Telli, MD^5^; Vered Stearns, MD^10^; Jerry S. Lanchbury, PhD^1^; Kirsten M Timms, PhD*^1^

**Affiliations:**

1. Myriad Genetics, Inc., Salt Lake City, UT, USA
2. Vanderbilt University Medical Center, Nashville, TN, USA
3. Mayo Clinic, Rochester, MN, USA
4. University of Rochester Medical Center, Rochester, NY, USA
5. Stanford University School of Medicine, Stanford, CA, USA
6. Northwestern University, Chicago IL, USA
7. University of Pennsylvania, Philadelphia, PA, USA
8. University of Texas Health Science Center at San Antonio, San Antonio, TX, USA
9. Duke University, Durham, NC, USA
10. Sidney Kimmel Comprehensive Cancer Center, Johns Hopkins School of Medicine, Baltimore, MD, USA
11. Melvin and Bren Simon Comprehensive Cancer Center, Indiana University School of Medicine, Indianapolis, IN, USA
12. Beth Israel Deaconess Medical Center, Boston, MA, USA
13. University of Washington, Seattle, WA, USA
14. Fred Hutchinson Cancer Research Center, Seattle, WA, USA
15. AstraZeneca, Cambridge, UK
16. AstraZeneca, Boston, MA, USA
17. Royal College of Surgeons in Ireland, Dublin, Ireland
18. Dana-Farber Cancer Institute, Boston, MA
19. Harvard Medical School, Boston, MA
20. Oregon Health & Science University, Portland, OR, USA
21. Cancer Research @UCC, University College Cork, Cork, Ireland

***Corresponding author:**

Kirsten M. Timms, PhD

320 Wakara Way

Salt Lake City, UT 84108

ktimms@myriad.com

# Supplemental Figure 1. Distribution of GIS in *BRCA*1-deficient and *BRCA*2-deficient samples by cancer type. GIS distributions for *BRCA1*-deficient and *BRCA2*-deficient samples were compared for each cancer type using Kolmogorov-Smirnov tests [samples with both *BRCA1* and *BRCA2* deficiencies were excluded from the comparisons (N = 1 ovarian sample and N = 1 TNBC sample)]. A significant difference between the GIS distribution of *BRCA1-*deficient and *BRCA2*-deficient samples was observed for TNBC (p=0.002) and ER+ breast cancer (p=0.02), but no significant difference was observed for ovarian cancer (p=0.167).


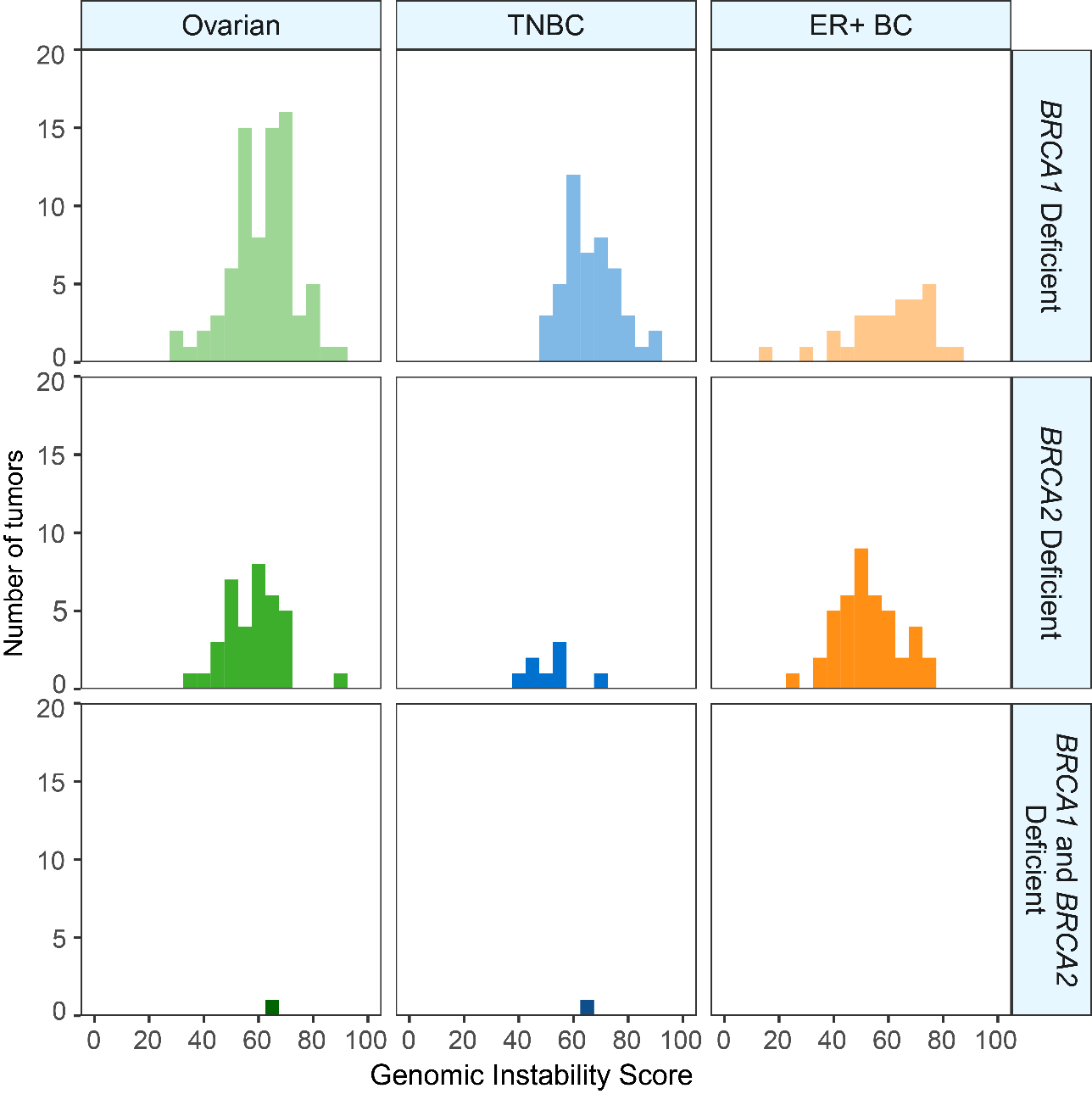


# Supplemental Figure 2. Distribution of genomic instability score (GIS) in *BRCA-*deficient tumors.
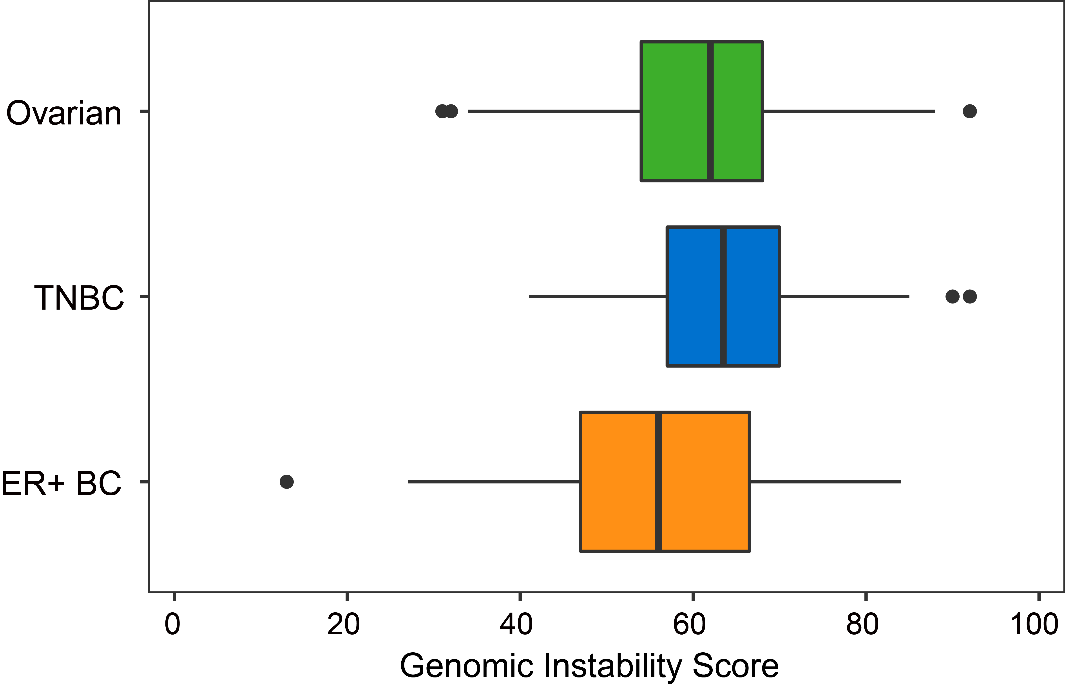


# Supplemental Figure 3. Distribution of genomic instability score (GIS) in ER+ breast cancer tumors with known HER2 status.


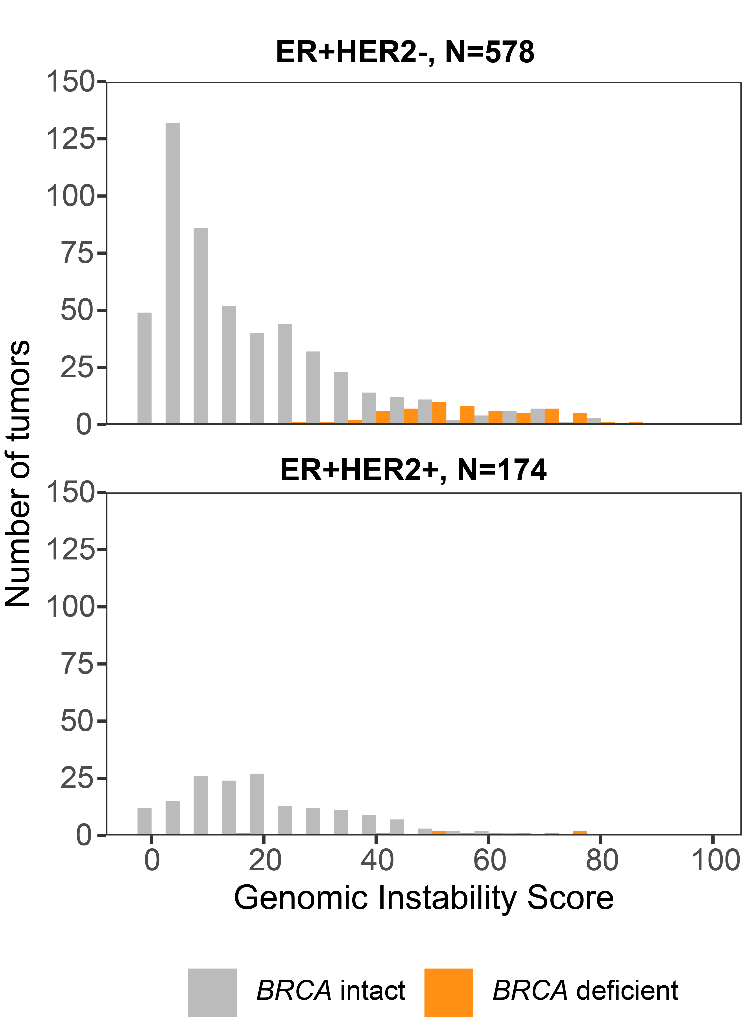


# Supplemental Figure 4. Odds ratios from univariable logistic regression models measuring the ability of Genomic Instability Score (GIS) threshold status to predict pathologic complete response (pCR) in triple negative breast cancer (TNBC).


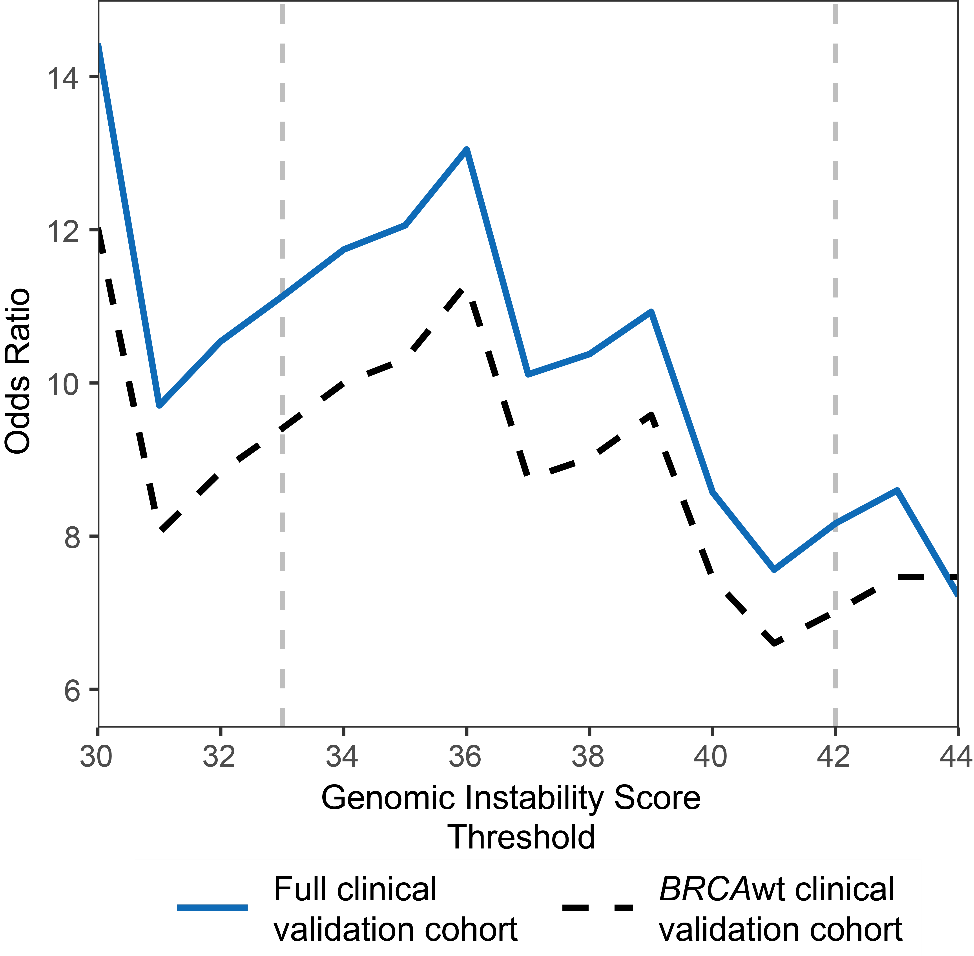


Models were fit in the full clinical validation cohort (N=211, solid line) and in the *BRCA*wt clinical validation cohort (N=171, dashed line). The solid and dashed lines represent the ability of GIS threshold status to predict pCR, calculated using odds ratios. The vertical grey dashed lines highlight GIS threshold statuses of $\boldsymbol{\geq}$33 and $\boldsymbol{\geq}$42.

# Supplemental Table 1. Rates of *BRCA*1 and *BRCA*2 deficiency by cancer type.

| **Cancer Type** | ***BRCA1* Deficient** | ***BRCA2* Deficient** | ***BRCA1* and *BRCA2* Deficient** |
| --- | --- | --- | --- |
| Ovarian | 78/115 (67.8%) | 36/115 (31.3%) | 1/115 (0.9%) |
| TNBC | 47/56 (83.9%) | 8/56 (14.3%) | 1/56 (1.8%) |
| ER+ | 29/71 (40.8%) | 42/71 (59.2%) | 0/71 (0%) |

Abbreviations: ER+, estrogen receptor positive; TNBC, triple negative breast cancer.
